# ELM-CLAIM: a claim-calibration framework for adaptive, memory-like, learning-like, and goal-directed claims in engineered living materials

**DOI:** 10.3389/fsysb.2026.1905585

**Published:** 2026-10-14

**Authors:** Jordi Vallverdú

**Affiliations:** Department of Philosophy, Universitat Autònoma de Barcelona, Bellaterra, Catalonia, Spain

**Keywords:** adaptive materials, attribution, biohybrid systems, claim calibration, engineered living materials, experimental controls, learning-like behavior, memory-like behavior

## Abstract

Engineered Living Materials (ELMs) combine living cells with matrices, interfaces, environmental fields, and sometimes electronic or computational controllers. Their multicomponent architecture creates an attribution problem: the same observed output may reflect passive material physics, active cellular regulation, interface or morphological change, environmental gradients, controller adaptation, or coupled-system dynamics. This article proposes ELM-CLAIM (Engineered Living Materials—Controls for Learning, Adaptation, Intelligence, and Memory), an author-proposed claim-calibration and reporting framework for matching adaptive vocabulary to measurable state variables, temporal criteria, attribution targets, and evidence classes. The framework classifies specific behaviors from passive responsiveness (L0) through active responsiveness (L1), adaptation (L2), memory-like behavior (L3), learning-like behavior (L4), and autonomous goal-directedness (L5). It also provides an evidence-tiered claim-to-control matrix, proportional substitution rules for experimentally intractable controls, component- and controller-level attribution logic, model-transparency requirements, and a reviewer-oriented reporting checklist. Essential entries denote evidential functions rather than a single mandatory assay, allowing justified orthogonal controls while requiring claims to be lowered when major alternatives remain unresolved. Published cases are used illustratively to stress-test interpretability rather than to validate the framework statistically. ELM-CLAIM calibrates language; it does not by itself validate an experiment, define cognition, or constitute a community standard. Community testing, inter-reviewer reliability assessment, and prospective experimental use remain necessary next steps.

## 1 Introduction

Engineered Living Materials (ELMs) are one of the clearest contemporary examples of convergence between materials science, synthetic biology, biofabrication, and systems engineering. In an ELM, living cells—bacteria, yeasts, mammalian cells, fungi, microbial consortia, organoid-like tissues, or engineered biofilms—are deliberately integrated with a material phase such as a hydrogel, polymer, mineral matrix, cellulose network, elastomer, textile, ceramic, implantable scaffold, or electronic interface. The resulting system can combine material structure with biological metabolism, growth, gene expression, collective organization, sensing, secretion, remodeling, and repair (Nguyen et al., 2018; Chen et al., 2015; Gilbert and Ellis, 2019; Srubar, 2021; Rodrigo-Navarro et al., 2021; Rivera-Tarazona et al., 2021).

The field has produced striking examples: living functional materials based on engineered biofilms (Chen et al., 2015); light-patterned bacteria on textiles, ceramics, and plastics (Moser et al., 2019); 3D-printed bacteria embedded in functional architectures (Schaffner et al., 2017); programmable and printable *Bacillus subtilis* biofilms (Huang et al., 2019); therapeutic matrix-forming *Escherichia coli* Nissle systems (Praveschotinunt et al., 2019); and biohybrid actuators linking living cells to robotic or device architectures (Ricotti et al., 2017; Raman et al., 2016; Raman and Bashir, 2017). More recent work has extended ELM fabrication toward practical manufacturing, mechanically tunable living materials, and explicitly adaptive or evolving cell–matrix designs (Lu et al., 2024; Manjula-Basavanna et al., 2024; Cui et al., 2026). These systems are not inert carriers of cells. They are coupled living–material systems in which cell state, scaffold chemistry, mechanics, interface geometry, environmental context, and external control may jointly determine system behavior.

As a result, ELM research increasingly uses language historically associated with adaptive systems and cognition. Materials are said to “sense,” “respond,” “adapt,” “remember,” “learn,” “decide,” or exhibit “autonomous” behavior (Nguyen et al., 2018; Rivera-Tarazona et al., 2021). Some of this vocabulary is scientifically useful. A bacterial genetic toggle switch can store a prior molecular input as a stable state (Gardner et al., 2000). Microbial regulatory networks can show predictive responses to correlated environmental changes (Tagkopoulos et al., 2008). Biofilms and cell-laden matrices can remodel material properties across time (Chen et al., 2015; Huang et al., 2019). Smart abiotic materials can also show hysteresis, shape memory, self-healing, and stimulus responsiveness (Hager et al., 2010; Behl and Lendlein, 2007). Therefore, the issue is not whether ELMs can exhibit functions that are legitimately history-dependent or adaptive. The issue is whether a specific claim is operationally defined, experimentally controlled, and attributed to the right system level.

Three recurring errors motivate ELM-CLAIM. First, attribution errors occur when an observed behavior is attributed to living cells although it arises from the scaffold, or attributed to the scaffold although it requires active cellular metabolism, interface remodeling, or controller feedback. Second, *scalar errors* occur when molecular bistability, single-cell physiological change, population selection, tissue-scale morphology, and whole-material performance are all called “memory” without specifying the scale at which the state is encoded. Third, reproducibility errors occur when one laboratory reports “learning” using criteria that another would classify as habituation, sensitization, selection, maturation, damage, fatigue, or controller tuning. These risks are amplified in ELMs because the systems are intentionally built: the investigator chooses the cell, scaffold, geometry, circuit, controller, and stimulus history. A capacity may therefore be designed into the material, emerge from living–material coupling, or merely appear at the output because an external algorithm performs the relevant adaptation.

ELM-CLAIM addresses this problem as a claim-calibration and attribution framework. It is not intended to settle philosophical debates about cognition, minimal agency, consciousness, or sentience (Lyon, 2006; Baluška and Reber, 2019; Levin, 2019). Instead, it provides operational language for authors, reviewers, and editors: what is being claimed, which measurements define the claim, which controls are needed, which component or coupling relation is responsible, and which claims remain speculative. The framework is designed to protect both rigor and innovation. It prevents cognitive vocabulary from becoming uncontrolled metaphor, but it also prevents legitimate adaptive functions from being dismissed merely because they occur outside nervous systems.

The article proceeds as follows. Section 2 defines the framework’s scope, derivation, and key terms. Section 3 situates ELM claims in relation to adjacent literatures on adaptive behavior, systems biology, synthetic biology standards, and smart materials. Section 4 proposes a six-level claim taxonomy. Section 5 introduces an evidence-tiered claim-to-control matrix. Section 6 develops attribution logic for multicomponent systems. Section 7 provides a minimal reporting checklist. Section 8 stress-tests the framework on published cases. Section 9 discusses limitations, extensions, and community implementation. Section 10 concludes.

## 2 Scope, definitions, and framework derivation

### 2.1 Scope of the framework

ELM-CLAIM applies to manuscripts that make adaptive, memory-like, learning-like, intelligent, autonomous, or goal-directed claims about engineered living materials or closely related biohybrid systems. The framework is relevant when at least one living component is intentionally coupled to at least one material, interface, environmental, computational, or device component, and when the claimed behavior depends on more than a single instantaneous measurement. It is equally applicable to microbial ELMs, biofilm materials, cell-laden hydrogels, morphogenetically active materials, ELM-device hybrids, and systems incorporating engineered gene circuits.

The framework does not require authors to use cognitive vocabulary. Rather, it tells authors what evidential commitments follow if such vocabulary is used. A paper may report an impressive cell-mediated material response without calling it memory or learning. Conversely, if authors call a behavior “memory-like” or “learning-like,” ELM-CLAIM specifies the minimum reporting and control logic needed for the claim to be intelligible and reproducible. These three origins and their claim consequences are summarized in Table 1.

**TABLE 1:** Origins of adaptive behavior distinguished by ELM-CLAIM.

Origin	Operational meaning	Claim consequence
Designed	The relevant state transition is explicitly installed by the investigator, for example, by a bistable genetic circuit or adaptive controller	Legitimate engineering achievement; label as designed memory or controller-mediated adaptation rather than emergent biological learning
Selected	Stimulus history changes the composition of a population by differential survival, growth, or phenotypic enrichment	May explain material-level change, but should not be called individual learning unless acquisition within cells or coupled units is demonstrated
Emergent	No isolated component reproduces the behavior; the behavior appears only through interaction among living cells, scaffold, interface, morphology, environment, or controller	Supports coupled-system attribution when component deletion or decoupling shows that interaction is necessary

Because “intelligence” is too broad to function as a primary operational category, ELM-CLAIM treats intelligence-like language as a composite or interpretive claim that must be decomposed into lower-level measurable claims: responsiveness, history dependence, persistence, acquisition, extinction, reference-state control, or coupled-system emergence. Thus, a manuscript should not simply claim that an ELM is intelligent; it should state which operational behaviors support that interpretation and which remain speculative.

### 2.2 Definition of behavior in ELM-CLAIM

A central ambiguity in the ELM literature is the word “behavior.” In ELM-CLAIM, a behavior is a measurable, stimulus- or context-dependent change in one or more state variables of an engineered living material over time. State variables may include mechanical properties, geometry, swelling, conductivity, permeability, optical output, chemical secretion, biofilm architecture, cell viability, gene expression, electrophysiological activity, metabolic flux, morphology, or controller state. A behavior therefore need not be movement or animal-like action. It is any reproducible input–state–output relation for which time, history, and system level can be specified.

A valid ELM-CLAIM statement has the following grammar:

*Under stimulus protocol* P *, system* S *exhibits behavior* B *, measured by state variable(s)* X *, satisfying claim level* $L_{n}$ *, attributed to component(s)* $C_{i}$ *or coupled system* C *, on the basis of controls* K .

This grammar makes clear that a claim is not simply a word such as “memory.” A claim specifies a measurable variable, temporal window, stimulus history, attribution target, and control set.

### 2.3 Framework derivation

ELM-CLAIM was developed as a problem-driven methodological synthesis rather than as a quantitative systematic review. The source set was assembled purposively and iteratively around five evidential functions required by the problem: (i) defining the architectures and claimed capabilities of engineered living and living functional materials (Nguyen et al., 2018; Chen et al., 2015; Gilbert and Ellis, 2019; Srubar, 2021; Rodrigo-Navarro et al., 2021; Rivera-Tarazona et al., 2021; Lu et al., 2024; Manjula-Basavanna et al., 2024; Cui et al., 2026); (ii) identifying how designed biological state and memory are implemented in synthetic circuits (Elowitz and Leibler, 2000; Gardner et al., 2000; Khalil and Collins, 2010; Voigt, 2020); (iii) recovering operational distinctions among habituation, conditioning, adaptation, and learning (Thorpe, 1963; Rescorla, 1988; Rankin et al., 2009); (iv) importing systems-biology principles of multiscale modeling, perturbation, feedback, identifiability, and model exchange (Kitano, 2002; Hucka et al., 2003; Waltemath et al., 2011; Bergmann et al., 2014; Villaverde et al., 2016); and (v) linking claims to reporting, reproducibility, and model-credibility practices (Taylor et al., 2008; Galdzicki et al., 2014; Wilkinson et al., 2016; Waltemath et al., 2011; Sauro et al., 2026).

Sources were included when they contributed at least one of four elements needed to operationalize a claim: a measurable state variable, a temporal criterion, a causal/attribution test, or a reproducibility/reporting rule. Sources were not selected merely because they used terms such as “intelligence” or “learning.” Conversely, work was excluded from the core derivation when it was purely metaphorical, lacked a material or biohybrid connection relevant to attribution, or did not add an operational criterion beyond sources already represented. Foundational sources were retained where the relevant criterion remains current; recent ELM and computational-modeling literature was added to check that the framework covers contemporary fabrication, adaptive cell–matrix coupling, and model/controller-rich systems. This strategy is intentionally transparent but not exhaustive: no PRISMA-style screening, bibliometric weighting, or claim of complete literature coverage is made.

The levels were derived by asking which additional evidential commitment is introduced when claim language becomes stronger. L0 requires only a current passive input–output relation; L1 adds necessary active living or biointerface participation; L2 adds dependence on stimulus history; L3 adds persistence after stimulus removal; L4 adds experience-dependent acquisition plus retention and modifiability with major confounds addressed; and L5 adds endogenous reference-state control under perturbation. The ordering is therefore an evidence ladder for a specified behavior, not a biological scala naturae and not a maturity ranking of whole materials.

Several alternative structures are possible. A binary adaptive/non-adaptive scheme was rejected because it collapses passive hysteresis, cellular regulation, and persistent state into one category. A shorter three-level scheme (responsive–history-dependent–goal-directed) was rejected because it hides the experimentally important boundary between adaptation, retained state, and learning-like acquisition. A fully mechanism-specific taxonomy was also rejected because mechanisms differ across microbial, mammalian, scaffold-dominated, and controller-coupled ELMs. The six-level structure is therefore a pragmatic compromise: few enough categories for reviewer use, but enough separation to make changes in temporal and causal evidence explicit. It should be revised if community testing shows that particular boundaries are unreliable.

The design goal was not to produce a philosophical definition of cognition, but to translate common adaptive-language claims into reviewer-actionable criteria. The resulting framework was constrained by four principles: (1) claims must be operationally measurable; (2) attribution must be component-resolved unless coupled-system emergence is demonstrated; (3) controls should be strong enough to prevent overclaiming but flexible enough for diverse ELM platforms; and (4) reporting should interoperate with existing standards rather than inventing an isolated checklist.

### 2.4 Designed, selected, and emergent adaptive behavior

ELM-CLAIM distinguishes three origins of adaptive behavior. Designed behavior is explicitly engineered, as when a bistable circuit is installed to store a prior input (Gardner et al., 2000). Selected behavior arises because subpopulations with different phenotypes expand or contract under stimulus history, producing population-level change without individual learning. Emergent behavior arises from interactions among living cells, scaffold, interface, morphology, environmental gradients, and controller dynamics that no isolated component exhibits alone. All three are scientifically legitimate, but they support different claims. Designed memory demonstrates engineering achievement; emergent memory demonstrates a system-level property; selection may mimic learning but requires different vocabulary unless individual or coupled-system acquisition is shown. These three origins and their claim consequences are summarized in Table 1.

## 3 Background: adaptive behavior in ELMs and the vocabulary problem

### 3.1 ELM architectures relevant to adaptive claims

ELMs vary in composition and therefore in attribution complexity. At least six architectural classes are relevant.

1. Cell-laden scaffolds: living cells are encapsulated in or seeded onto a preformed matrix such as a hydrogel, polymer, or extracellular-matrix mimic (Tibbitt and Anseth, 2009).
2. Cell-secreted materials: cells produce the matrix itself, as in biofilm or bacterial cellulose systems (Chen et al., 2015; Moser et al., 2019; Huang et al., 2019).
3. Genetically programmed ELMs: cells carry engineered circuits that switch, compute, secrete, pattern, or record inputs (Gardner et al., 2000; Khalil and Collins, 2010; Voigt, 2020).
4. Morphogenetically active ELMs: cell growth, migration, contraction, differentiation, or collective organization changes material shape or function (Raman and Bashir, 2017; Ricotti et al., 2017).
5. Therapeutic or environmental living materials: living cells are embedded in matrices to deliver functions *in vivo* or in ecological settings (Praveschotinunt et al., 2019; Rivera-Tarazona et al., 2021).
6. ELM-device hybrids: living material components interact with sensors, actuators, electronics, optics, microfluidics, or adaptive algorithms.

Because these architectures combine multiple causal layers, the same output may have different explanations. Progressive stiffening may be passive viscoelastic hysteresis, active matrix deposition, cell-mediated remodeling, selection of a subpopulation, structural consolidation of the scaffold, or feedback from an external controller. ELM-CLAIM is built to discriminate among these alternatives.

### 3.2 Why systems biology matters

ELM adaptive claims are systems-biology claims whenever they involve feedback, multiscale state changes, emergent dynamics, or causal attribution across levels. A systems-biology perspective asks not only whether an output changes, but how state variables interact across scales, how perturbations propagate, whether the system has attractors or hysteresis, whether parameters are identifiable, and whether the model explaining the behavior is reproducible (Kitano, 2002; Hucka et al., 2003; Waltemath et al., 2011).

This matters because adaptive vocabulary is often a proxy for dynamic structure. “Memory” implies that present behavior depends on past states. “Learning” implies acquisition, retention, and modification of future responses by experience. “Autonomy” implies internally maintained organization rather than mere external control. These are not merely semantic labels; they are hypotheses about state-space trajectories, feedback architecture, causal sufficiency, and time-dependent perturbation response. ELM-CLAIM therefore treats adaptive vocabulary as a commitment to a causal and dynamical model, even when that model is qualitative.

### 3.3 Lessons from adjacent fields

ELM research can draw from several adjacent debates. Behavioral neuroscience and comparative psychology developed operational criteria for habituation, sensitization, conditioning, and learning because surface behavior alone was insufficient to infer mechanism (Thorpe, 1963; Rescorla, 1988; Rankin et al., 2009). Synthetic biology developed standard languages and model-sharing practices because genetic designs, circuit diagrams, and computational models must be communicated in machine-readable and reproducible ways (Galdzicki et al., 2014; Voigt, 2020). Systems biology developed standards for model exchange and simulation reproducibility, including SBML, MIASE, and COMBINE archives (Hucka et al., 2003; Waltemath et al., 2011; Bergmann et al., 2014). Biomedical reporting initiatives such as MIBBI and FAIR principles emphasize that reproducibility requires more than narrative description; it requires standardized minimum information and reusable data (Taylor et al., 2008; Wilkinson et al., 2016).

ELM-CLAIM imports this standards logic into living materials. Its core assumption is modest: if a material is described using adaptive or cognitive-like language, the claim should be tied to explicit operational variables, controls, attribution criteria, and reproducibility information.

## 4 The ELM-CLAIM claim taxonomy

The ELM-CLAIM taxonomy classifies claims, not systems. A single ELM may support an L1 claim for one output, an L3 claim for another output, and no adaptive claim for a third. Levels L0–L5 should therefore be read as evidential thresholds for specific behaviors rather than as fixed ontological ranks of materials.

Two dimensions organize the taxonomy. The first is temporal dependence: does the behavior depend only on current stimulus conditions, or also on prior stimulus history? The second is active system participation: can the behavior be explained by passive physical chemistry, or does it require living metabolism, gene regulation, biointerface activity, morphology, controller feedback, or coupled-system organization? Figure 1 summarizes the two-dimensional organization of the six claim levels.

**FIGURE 1:**
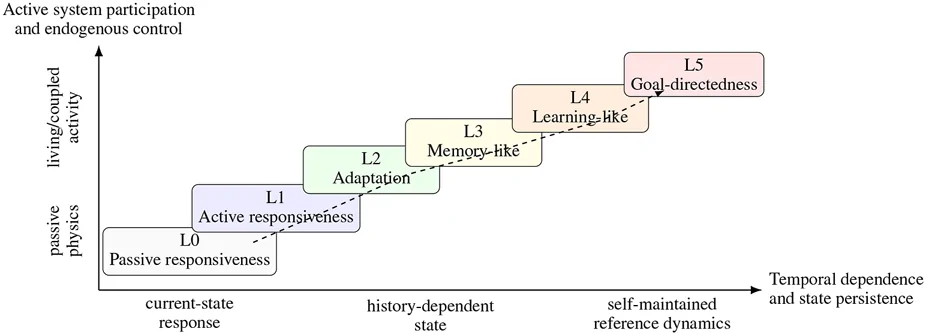
Two-dimensional organization of ELM-CLAIM claim levels. The taxonomy classifies evidence for specific measured behaviors, not the maturity or value of whole systems; a rigorously demonstrated L1 claim can be stronger science than an unsupported L4 or L5 claim.

### 4.1 Operational boundary rules

The taxonomy deliberately avoids universal numerical cutoffs because ELM platforms differ in assay noise, timescale, and physically meaningful effect size. It nevertheless requires explicit decision rules. First, terms such as “absent” or “strongly reduced” should mean that the relevant contrast is larger than the assay’s declared resolution and uncertainty and is biologically or materially meaningful under a pre-specified criterion; a small statistically significant difference is not automatically sufficient. Authors should report replicate structure, uncertainty, and limits of detection or quantification when these are applicable to the assay.

Second, history dependence (L2) means that two groups exposed to different prior stimulus histories produce different responses under the same current test condition. A time trend alone is insufficient. Third, persistence (L3) requires evidence after the inducing stimulus has been removed or returned below its effective range and after the expected immediate relaxation of an L0/L1 response. The retention interval must be declared relative to system kinetics. At minimum, authors should include a time-matched naive control and a retained post-stimulus measurement; a multi-point decay or state trajectory is strongly preferred and is essential when the relaxation timescale is unknown.

Fourth, specificity means a pre-specified contrast showing that the retained or acquired change is not an indiscriminate consequence of damage, generalized stress, or drift. This may be tested with a related but distinct stimulus, an unpaired protocol, a transfer test, or another architecture-appropriate contrast. Finally, “cannot be explained by” is not a claim of logical proof. It means that the major plausible alternatives for the specified system have been directly tested, orthogonally constrained, or identified as residual uncertainty. If a major alternative remains untested, the claim should be lowered or explicitly marked provisional. These rules operationalize the L2–L4 boundaries without pretending that one numerical threshold is valid for every ELM.

### 4.2 Level 0: passive responsiveness

Definition. A change in material properties or system state that is a direct, immediate, reversible, and physically or chemically expected consequence of an environmental stimulus, requiring no active cellular contribution.

Examples. Hydrogel swelling under pH change, thermal expansion, photochromic absorbance change, passive diffusion, viscoelastic hysteresis, or shape-memory behavior in an acellular material.

Appropriate vocabulary. Responsive, stimulus-sensitive, physically reactive, passive material response.

Inappropriate vocabulary. Adaptive, memory-like, learning-like, intelligent, goal-directed.

Core evidence. The behavior is reproduced by an acellular or killed-cell control prepared and stimulated in the same way. If the acellular system reproduces the behavior within measurement error, the claim remains L0 even if living cells are present in the experimental material.

### 4.3 Level 1: active responsiveness

Definition. A change in material properties or system state that requires metabolically active living cells, active biointerface processes, or engineered biological regulation, but does not depend on prior stimulus history.

Examples. Quorum-sensing activation of a biofilm output, strain-induced reporter expression, pH-dependent peptide secretion, light-induced living patterning, or cell-mediated matrix deposition under current stimulus.

Appropriate vocabulary. Biologically active responsiveness, cell-mediated responsiveness, dynamically regulated output.

Core evidence. The response is absent or materially reduced, under the pre-specified assay-resolution rule in Section 4.1, in acellular or viability-controlled inactive-cell conditions, and the relevant cellular state is measured independently of the output variable.

### 4.4 Level 2: adaptation

Definition. A history-dependent change in response magnitude, kinetics, threshold, or qualitative output caused by prior exposure to the same or a related stimulus, without necessarily requiring persistence across long gaps. Three subtypes should be specified.

1. L2a—Habituation: response decreases after repeated, non-reinforced presentation of a stimulus and can recover after rest (Rankin et al., 2009).
2. L2b—Sensitization: response increases after prior strong, novel, or damaging stimulation.
3. L2c—Associative adaptation: response to stimulus B changes after paired presentation with stimulus A, compared with unpaired and backward controls (Rescorla, 1988; Tagkopoulos et al., 2008).

Appropriate vocabulary. Adaptive, habituating, sensitizing, associative adaptation, history-dependent response. The subtype must be named.

Core evidence. Stimulus-history controls distinguish genuine adaptation from drift, maturation, fatigue, damage, selection, and environmental depletion.

### 4.5 Level 3: memory-like behavior

Definition. A persistent state change that outlasts the inducing stimulus and alters later behavior after a stimulus-free interval. L3 claims require a declared persistence interval and a measurable state variable.

1. L3a—Short-term memory-like: persistence on signaling or metabolic timescales, such as minutes to hours.
2. L3b—Long-term cellular memory-like: persistence through stable molecular, epigenetic, transcriptional, proteomic, metabolic, or cytoskeletal states across longer intervals or cell divisions.
3. L3c—Structural memory: persistence encoded primarily in scaffold or interface architecture, such as remodeled matrix, altered porosity, mineral deposition, or mechanically consolidated biofilm structure.
4. L3d—Designed circuit memory: persistence implemented by an engineered circuit, such as a bistable switch. This is a valid memory-like claim but should be explicitly labeled as designed memory.

Core evidence. The inducing stimulus is shown to be removed or returned below its effective range; persistence is then demonstrated over a declared interval relative to the system’s relaxation kinetics, with time-matched naive controls. A multi-point post-stimulus trajectory is strongly recommended (and expected when kinetics are unknown), but a smaller number of measurements may be justified when the relevant state is known to be stable and the retention interval is mechanistically motivated. Specificity is tested with an appropriate related-stimulus or alternative-history contrast. The encoding substrate—cellular, scaffold, interface, controller, or coupled system—is identified.

### 4.6 Level 4: learning-like behavior

Definition. A process by which defined experience produces a persistent, specific, and modifiable change in subsequent ELM behavior that cannot be explained by maturation, development, selection, fatigue, damage, simple hysteresis, or external controller training alone.

A full L4 claim requires four classes of evidence.

1. Acquisition: the behavioral modification is absent before the training protocol and appears after controlled exposure.
2. Retention: the modification persists beyond the stimulation period, thereby satisfying an L3 criterion for the relevant state variable.
3. Specificity or transfer: the modification is stimulus-specific or generalizes to a defined related stimulus in a way predicted before testing.
4. Reversibility or extinction: the modification can be reduced, abolished, or overwritten by a defined subsequent protocol.

Because ELMs differ radically in timescale and output modality, ELM-CLAIM also allows L4-provisional wording, but only under a restricted condition: acquisition and retention are demonstrated, maturation/fatigue/damage/selection and controller-only learning are explicitly addressed, and transfer or extinction are technically unavailable or scientifically undefined for the system. L4-provisional is not equivalent to full L4. It is a constrained reporting label, and the manuscript should state exactly: “learning-like under acquisition and retention criteria; transfer/extinction not established.” Authors should not state an unqualified learning-like claim unless all four evidence classes above are met for the same measured behavior.

### 4.7 Level 5: autonomous goal-directedness

Definition. System-level behavior in which an ELM maintains or recovers a biologically generated reference condition under perturbation by closed-loop organization that cannot be reduced to a fixed external set point, simple stimulus–response mapping, or controller-only optimization.

L5 is deliberately stringent. Homeostatic feedback alone is not sufficient if the target value is imposed entirely by the investigator or by an external controller. A legitimate L5 claim requires: (i) an explicit architecture of sensing, comparison, action, and feedback; (ii) perturbation of the putative reference state; (iii) manipulation showing that the reference condition is endogenously generated or maintained by the living/coupled system; and (iv) performance under at least one perturbation not used in the design or training protocol. The cybernetic literature establishes that goal-directed language requires more than directional response; it requires reference-dependent control under perturbation (Rosenblueth et al., 1943; Ashby, 1956).

A minimal L5 statement should therefore report five elements: the reference variable r or reference region R ; the measured system state x(t) ; the comparator or deviation-detection process that evaluates x(t)−r or departure from R ; the actuator process that changes cellular, material, morphological, or controller state; and the perturbation protocol that displaces the system from the reference condition. The decisive evidence is not return-to-set-point alone, but return mediated by a living or coupled architecture whose reference condition can be perturbed, manipulated, and distinguished from an externally imposed software or experimental parameter.

## 5 The evidence-tiered claim-to-control matrix

The original purpose of a claim-to-control matrix is to make explicit which controls are needed to support which claim. However, ELMs are experimentally diverse. A genetic knockout may be essential in a tractable microbial system but impossible in a primary mammalian-cell hydrogel. Cell-free biochemical reconstitution may be decisive for an enzyme-mediated response but unrealistic for a multicellular morphogenetic material. ELM-CLAIM therefore uses an evidence-tiered matrix rather than a universal binary checklist.

- E = Essential: normally required for the claim. If absent, the claim should be lowered or explicitly labeled provisional.
- R = Recommended: strongly strengthens attribution but may be replaced by a justified alternative.
- A = Alternative/system-specific: appropriate in some ELM classes; authors should explain why the selected alternative is adequate.

### 5.1 Normative force, minimum evidence, and proportional substitution

The matrix specifies evidential functions, not a universal list of assays. An E entry means that the causal alternative represented by that entry normally must be addressed for the stated claim level. It does not mean that every laboratory must perform the same knockout, inhibitor, recellularization, or long-duration protocol. An R entry is best practice that strengthens attribution; an A entry identifies a family of architecture-dependent options. Reviewers should therefore ask whether the evidential function has been met, not whether a favored technique has been used.

A laboratory that cannot perform an ideal perturbation should follow a three-step substitution rule: (1) state why the ideal control is technically or biologically infeasible; (2) use an orthogonal control, natural experiment, minimal reconstitution, temporal contrast, or model-based perturbation that addresses the same alternative explanation as directly as possible; and (3) report the residual uncertainty created by the substitution. For example, if a genetic knockout is impossible in a primary-cell material, viability-controlled inhibition plus an independent readout of the implicated pathway may address the same necessity question. If no credible substitute addresses an essential causal alternative, the appropriate response is to lower the claim or use provisional wording rather than to penalize the system for being experimentally difficult.

The minimum acceptable evidence consequently scales with claim strength. L0 requires a matched demonstration that passive/acellular material can account for the response. L1 requires evidence that active living or biointerface function is necessary. L2 additionally requires a controlled stimulus-history contrast. L3 adds post-stimulus retention with a declared interval and time-matched control. L4 adds acquisition, retention, specificity/transfer or a justified provisional boundary, and explicit treatment of major confounds. L5 adds an annotated feedback architecture, reference-state perturbation, and evidence that the reference condition is generated or maintained by the living/coupled system. Recommended and alternative entries should not be treated as cumulative mandatory experiments.

### 5.2 System component ontology

The attribution target must be declared using the following component ontology, shown schematically in Figure 2:

1. L—Living cells: metabolism, viability, growth, gene expression, cell signaling, secretion, motility, electrophysiology, cytoskeleton.
2. S—Scaffold: abiotic matrix chemistry, mechanics, porosity, topography, conductivity, swelling, degradation, diffusion, hysteresis.
3. B—Biointerface: adhesion, mechanotransduction, surface chemistry, membrane–material coupling, cell–matrix signaling.
4. M—Morphology: emergent geometry from growth, contraction, aggregation, patterning, matrix deposition, or cell–scaffold organization.
5. E—Environmental gradients: imposed fields, nutrient gradients, oxygen, pH, light, temperature, chemical stimuli, mechanical loading.
6. A—Algorithm/controller: external software, electronics, closed-loop control, machine-learning model, adaptive device parameters.
7. C—Coupled system: behavior requiring interaction among two or more components and not reproduced by any isolated component.

**FIGURE 2:**
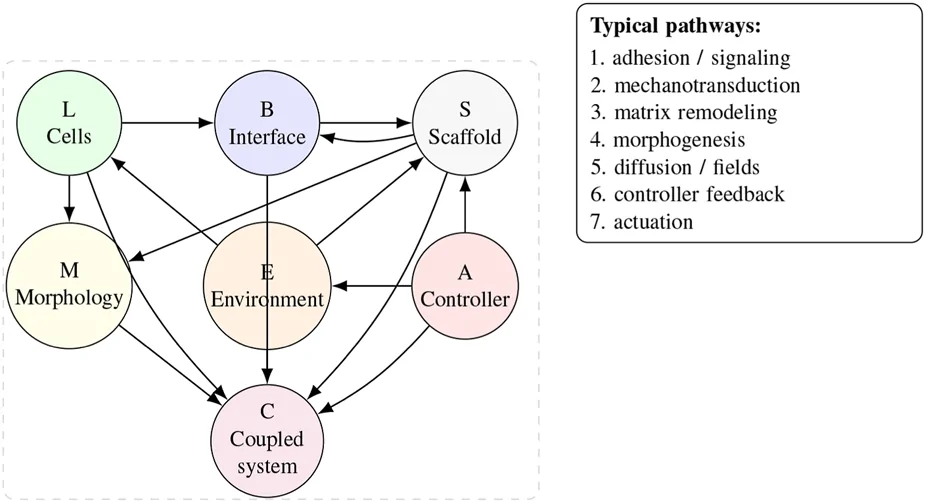
Component ontology for attribution in ELM-CLAIM. Arrows indicate causal direction among living cells, scaffold, interface, morphology, environment, controller, and coupled-system behavior. The pathway legend lists common mechanisms; actual manuscripts should report the architecture specific to their system rather than treating this schematic as universal.

### 5.3 Matrix

The evidence tiers and associated control/reporting requirements are summarized in Table 2.

**TABLE 2:** Evidence-tiered ELM-CLAIM matrix. E = essential, R = recommended, A = alternative/system-specific. The table should be interpreted with the claim grammar in Section 2; controls should be matched to the specific measured behavior and system architecture.

Claim	Likely attribution	Evidence tier	Controls and reporting requirements
L0 passive response	S or E	E	Acellular or killed-cell material reproduces the response under identical stimulus protocol; stimulus-off control; matched measurement timing
L0 passive response	S or E	R/A	Dose–response and hysteresis characterization; scaffold-only aging control; environmental-gradient mapping
L1 active response	L, B, or M	E	Acellular scaffold does not reproduce the response; inactive-cell or viability-controlled inhibition reduces the response; cell viability/state measured independently
L1 active response	L, B, or M	R/A	Genetic perturbation, pathway inhibitor with rescue, non-adhesive cell/control scaffold, decellularized conditioned scaffold, purified-factor reconstitution when mechanistically appropriate
L2a habituation	L, B, M, or C	E	Repeated-stimulus protocol; randomized inter-stimulus-interval or order control; recovery/rest test; exclusion of depletion, fatigue, and damage
L2b sensitization	L, B, M, or C	E	Sensitizing-stimulus-alone and test-stimulus-alone groups; time-matched naive controls; dose and damage assessment
L2c associative adaptation	L or C	E	Paired, unpaired, and backward-control protocols; pre-specified response variable; control for non-associative sensitization
L3 memory-like	L, S, B, M, A, or C	E	Persistence measured after stimulus removal at justified time points; naive time-matched controls; declared retention interval; specificity test
L3 memory-like	L, S, B, M, A, or C	R/A	Washout, re-challenge, consolidation inhibitor, recellularization of conditioned scaffold, single-cell lineage tracking, structural mapping, or controller-state audit depending on attributed substrate
L4-provisional	C, L, or A	E	Acquisition and retention demonstrated; maturation, selection, fatigue, damage, and controller-only learning excluded; training and testing protocols separated; manuscript states that transfer/extinction are not established and avoids unqualified learning-like language
L4 learning-like	C, L, or A	E	Acquisition, retention, specificity or transfer, and reversibility/extinction demonstrated for the same measured behavior; confounds above excluded
L4 learning-like	C, L, or A	R/A	Savings/reacquisition assay; single-cell or subpopulation analysis; controller-state audit; independent replication of the training/testing protocol where relevant
L5 autonomous goal-directedness	C, L, or A	E	Annotated feedback architecture; declared reference variable; comparator/deviation-detection process; actuator process; reference-state perturbation and manipulation; evidence that the reference condition is generated or maintained by the living/coupled system rather than imposed externally
L5 autonomous goal-directedness	C, L, or A	R/A	Novel perturbation not used in design/training; controller ablation; sensor/actuator decoupling; energetic and material-budget analysis

### 5.4 Decision procedure

ELM-CLAIM should be applied from lower to higher claims. Authors should first ask whether the behavior is reproduced by acellular or inactive controls. If so, L0 is the highest supported claim. If not, they should determine whether active living participation is necessary (L1). Only after current-state response is established should authors test history dependence (L2), persistence after stimulus removal (L3), acquisition/retention with exclusion of confounds (L4), or endogenous reference-state maintenance (L5). When controls are missing, the claim should be reduced or labeled provisional. Figure 3 summarizes this lower-to-higher decision procedure.

**FIGURE 3:**
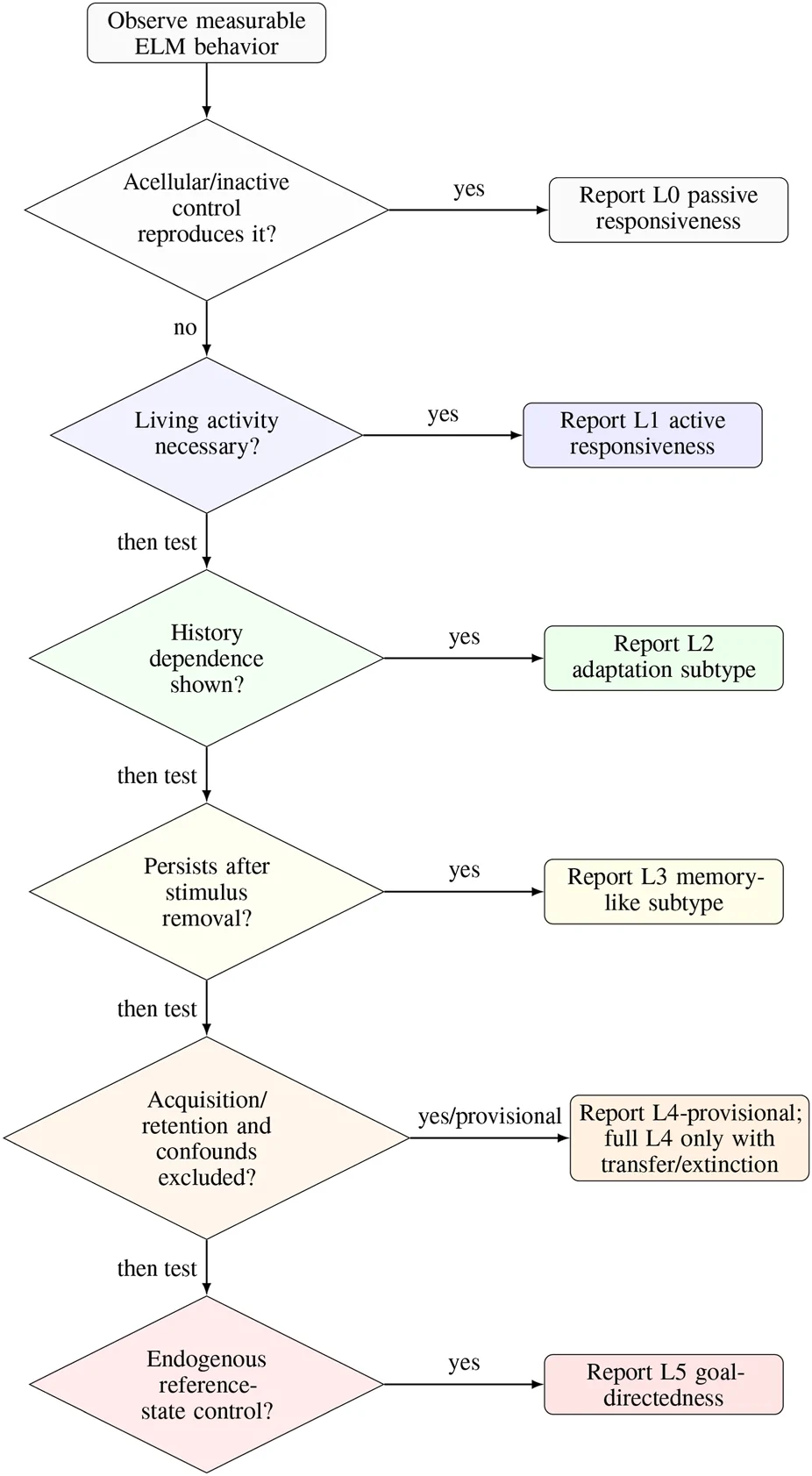
ELM-CLAIM decision flowchart. The decision procedure prevents direct jumps from evocative vocabulary to high-level claims without lower-level controls.

## 6 Attribution criteria for multicomponent ELMs

### 6.1 Principle of minimal attribution

ELM-CLAIM adopts the Principle of Minimal Attribution: an observed behavior should be attributed to the simplest component or coupling relation sufficient to explain it, given the evidence. Higher-level attributions must be earned by ruling out lower-level alternatives. This is not reductionism; it is a methodological guardrail. If a scaffold-only control explains a response, a cellular memory claim is unsupported. If living cells are necessary but the isolated cell population shows the same behavior, a coupled-system attribution is unnecessary. If neither isolated cells nor scaffold reproduce the behavior, and deletions of either component abolish it, coupled-system attribution becomes appropriate.

### 6.2 Necessary, sufficient, and emergent attribution

For a component-level attribution, authors should evaluate three questions.

1. Necessity: Does removing, disabling, inhibiting, or decoupling the component abolish or significantly reduce the behavior?
2. Sufficiency: Does the component reproduce the behavior alone, in a minimal reconstituted system, or in a scaled proxy model?
3. Specificity: Does perturbing plausible alternative components leave the behavior unchanged or change it in predicted ways?

A behavior should be attributed to the coupled system (C) when the full ELM shows the behavior but isolated components do not, and when component-deletion or interface-disruption experiments identify a necessary coupling architecture. Coupled-system attribution should not be used as a vague residual category. It requires evidence that interaction matters.

### 6.3 External controller confounds

ELM-device hybrids create a special risk: the living material may function as a sensor, actuator, or substrate while adaptation occurs entirely in software or electronics. Authors should therefore declare whether the controller is fixed, adaptive, trained offline, trained online, or closed-loop. A learning-like claim about the ELM is not supported if only the controller changes its parameters. In that case the correct statement is: “the ELM-device system exhibits controller-mediated adaptive performance.” If living dynamics and controller updates are both necessary and neither alone suffices, a coupled L4 or L5 claim may be considered. Recent biohybrid proposals that combine living systems, machine learning, real-time models, and adaptive control make this separation increasingly important (Carbonell et al., 2026).

### 6.4 Genetic circuit confounds

Bistable and multistable circuits can implement memory by design (Gardner et al., 2000). ELM-CLAIM treats this as scientifically valuable but requires explicit labeling. Authors should distinguish: (i) circuit memory in individual cells; (ii) population-level memory measured as a fraction of cells in a state; (iii) material-level memory where circuit state changes material function; and (iv) emergent memory where persistence arises from cell–material coupling beyond the circuit itself.

### 6.5 Population-level and selection confounds

Population-level changes may reflect individual adaptation, collective dynamics, or selection among pre-existing phenotypes. For L2 and above, authors should report whether evidence is single-cell, clonal, population-averaged, or material-level. Where feasible, lineage tracing, flow cytometry, single-cell reporters, spatial transcriptomics, or microcolony-level imaging should be used to distinguish cellular acquisition from selective enrichment. If selection is the dominant explanation, vocabulary should reflect population selection rather than learning-like modification.

### 6.6 Model and mechanism transparency

When adaptive claims depend on a mathematical or computational model, the model’s role should first be declared. A descriptive model summarizes observed trajectories or associations; a predictive model is evaluated on observations not used for fitting; a mechanistic model represents hypothesized causal state transitions and should generate perturbation-sensitive predictions. These roles are not interchangeable. A good descriptive fit does not by itself establish the mechanism or attribution required for an L3–L5 claim.

For quantitative models, authors should report equations or executable model structure, state variables, parameters, initial and boundary conditions, simulation protocol, parameter-fitting procedure, objective functions, and the data used for calibration versus evaluation. Structural or practical identifiability should be assessed when parameter-level interpretation matters, because unidentifiable parameters can support apparently good fits while leaving mechanistic conclusions underdetermined (Villaverde et al., 2016). Sensitivity analysis should identify which parameters or states materially control the claim; uncertainty from measurements, parameter estimation, stochasticity, and model structure should be propagated to the reported output where feasible.

Validation should be matched to the model’s context of use. A useful hierarchy is: code/implementation verification; calibration to designated data; evaluation on held-out conditions; prediction under a perturbation not used for fitting; and, for strong mechanistic claims, independent or cross-platform replication. Recent systems-biology guidance similarly emphasizes credibility, understandability, reproducibility, extensibility, validation, and uncertainty quantification (Sauro et al., 2026). Models should be deposited in standard formats where possible, such as SBML for biochemical networks and COMBINE/OMEX packages for multi-file modeling projects (Hucka et al., 2003; Bergmann et al., 2014); simulation protocols should satisfy MIASE principles (Waltemath et al., 2011).

For data-driven controllers and digital-twin architectures, authors should report which variables update online, what data are assimilated, which parameters are fixed or learned, update frequency, training/test separation, and whether the model itself closes the control loop. A digital twin can improve monitoring and prediction but is not evidence that the living material is adaptive or goal-directed: that attribution still requires component-resolved state changes and the controller diagnostics in Table 3. If models are qualitative, diagrams should identify feedback signs, measured and hidden variables, competing causal explanations, and perturbation predictions.

**TABLE 3:** Diagnostic guide for controller attribution in ELM-device hybrids.

Configuration	What changes during the experiment?	Default attribution	Claim guidance
Fixed controller	No controller parameters update	Living/material response or coupled fixed-loop behavior	Do not attribute adaptation to software; test whether living or material state changes with history
Offline-trained controller	Controller was optimized before the ELM experiment, then frozen	Designed controller contribution plus measured living/material response	Report training provenance; offline optimization is not evidence that the ELM learns during testing
Online adaptive controller	Software/electronic parameters update from incoming data	Controller-mediated adaptation unless living-state change is independently required	Audit parameter updates and compare with a frozen-controller condition before assigning L2–L4 to the ELM.
Living adaptive component with fixed controller	Living/material state changes with history while controller is fixed	Living component or coupled system, depending on necessity/sufficiency tests	L2–L4 may be considered if temporal criteria and confound controls are met.
Co-adaptive living–controller loop	Both living/material and controller states change and neither alone reproduces performance	Coupled system (C)	Report both state trajectories and decoupling tests; L5 additionally requires endogenous reference-state evidence, not merely closed-loop optimization

## 7 The ELM-CLAIM minimal reporting checklist

Table 4 provides a reviewer-ready checklist. The checklist is not a bureaucratic appendix; it defines the minimum reporting fields relevant to the claimed level and system architecture. Items that are genuinely not applicable should be marked as such with a brief rationale rather than treated as missing data. The checklist can be used by authors before submission, by reviewers during evaluation, and by editors when deciding whether a claim should be strengthened, softened, or marked provisional. A machine-readable CSV version is supplied as Supplementary Table S2.

**TABLE 4:** ELM-CLAIM minimal reporting checklist.

**Item**	**Requirement**	**What must be reported**	**Where**
A1	Claim level	L0–L5 and subtype; L4-provisional explicitly marked as not full L4 when transfer/extinction are absent	Abstract/Results
A2	Operational definition	State variable, decision threshold, assay resolution/LOD/LOQ when applicable, stimulus protocol, time window, retention interval	Methods/Results
A3	Vocabulary audit	Terms used in methods/Results match demonstrated level; speculation labeled	Whole manuscript
B1	System components	Cell type/strain, scaffold, interface, morphology, environment, controller	Methods
B2	Living state	Viability, growth, metabolic state, phenotype, engineered circuit state	Methods/Results
B3	Scaffold state	Composition, mechanics, porosity/topography, chemistry, aging, diffusion limits	Methods/Results
B4	Interface/morphology	Adhesion/coupling evidence, spatial organization, geometry, imaging or mapping	Results
B5	Controller	Algorithm, fixed/offline-trained/online-adaptive status, training/test separation, update rules, hardware/software versions	Methods
B6	Model role	Descriptive/predictive/mechanistic role; calibration/evaluation data; identifiability, sensitivity, and uncertainty as applicable	Methods/Supplement
C1	L0 control	Acellular or inactive-cell comparison under matched stimulus	Results
C2	L1 control	Evidence that active living/biointerface function is necessary using a viability-controlled or orthogonal perturbation	Results
C3	L2 control	Randomized, unpaired, backward, or time-order controls as subtype requires	Results
C4	L3 control	Stimulus removal/return below effective range; retained post-stimulus state; time-matched naive control; declared retention interval; specificity contrast	Results
C5	L4 control	Acquisition, retention, transfer or specificity, extinction/overwrite status, and maturation, fatigue, damage, selection, and controller-only confounds	Results
C6	L5 control	Reference variable, comparator, actuator, reference-state perturbation/manipulation, and feedback architecture	Results
C7	Feasibility/substitution	Infeasible ideal controls, substitute evidential function, and residual uncertainty	Methods/Discussion
D1	Attribution statement	“Behavior B is attributed to component(s) X on the basis of controls Y″	Discussion
D2	Alternatives ruled out	Scaffold, cell, interface, morphology, environment, controller alternatives addressed	Discussion
D3	Designed vs emergent	Explicit classification for engineered circuits and adaptive controllers	Results/Discussion
E1	Replication	Biological replicates, technical replicates, temporal repeats, failed runs	Methods
E2	Data and code	Raw data, processed data, code, model files, analysis notebooks	Data availability
E3	Standards	SBOL for genetic designs; SBML, COMBINE, and MIASE for applicable models	Methods/Supplement
E4	FAIR/CURE availability	Persistent identifiers, metadata, license, repository, software versions, and model credibility/reuse information when applicable	Data availability
E5	Machine-readable checklist	Completed checklist and case-audit fields supplied in CSV/JSON/XLSX or equivalent reusable format	Supplement

Reviewers can use Table 4 as a claim-calibration instrument. The recommended procedure is: identify the strongest adaptive word used in the Abstract, Results, or title; locate the measured behavior and state variable; assign the highest ELM-CLAIM level supported by the reported controls; request either additional controls, lower-level wording, or explicit provisional wording when evidence is incomplete. This procedure evaluates the claim language, not the scientific value of the material itself.

## 8 Stress-testing ELM-CLAIM on published cases

To illustrate usability, Table 5 applies ELM-CLAIM retrospectively to representative published systems. The cases were selected purposively rather than statistically: they span an abiotic boundary case, active and printed ELMs, designed circuit memory, predictive microbial regulation, biohybrid actuation, a closed-loop neural/controller edge case, and a review-level class of stimuli-responsive ELMs. Selection therefore maximizes diversity of attribution problems rather than representativeness of the literature. The examples include both ELM-core and adjacent systems because boundary cases are useful for testing whether the framework forces the analyst to state what is—and is not—being attributed.

**TABLE 5:** Concise retrospective application of ELM-CLAIM. The examples are illustrative rather than systematic and are intended to calibrate claim language across abiotic memory, active living function, controller-mediated behavior, and learning-like claims.

Case	ELM-CLAIM reading	Calibrated claim language	Evidence still needed for stronger claim
Shape-memory polymers (Behl and Lendlein, 2007)	L0 abiotic boundary case; not an ELM adaptive claim	“Shape-memory” or “hysteretic smart material”; not living memory or learning	A living component is absent; no metabolic, cellular, or coupled-system attribution is possible
Living functional materials based on engineered biofilms (Chen et al., 2015)	L1 active responsiveness/designed functionality	“Cell-mediated, genetically programmed material functionality”	Stimulus-history protocols, retention after stimulus removal, and attribution of state encoding
3D printing of bacteria into functional complex materials (Schaffner et al., 2017)	L1 active responsiveness; possible L3c only with retention and structural attribution	“Living printed material with cell-mediated function”	Acellular scaffold comparison after conditioning, recellularization, structural mapping, and retention assay
Programmable printable *B. subtilis* biofilms (Huang et al., 2019)	L1 designed active responsiveness; L3d only if stable circuit states are shown	“Programmable active living material”; specify designed vs. emergent persistence	Retention interval, circuit-state stability, material-level output persistence, and designed/emergent attribution
Genetic toggle switch in *E. coli* (Gardner et al., 2000)	L3d designed circuit memory at molecular/cellular level	“Designed circuit memory”	Coupling to material function, single-cell/population attribution, switching error rates, and retention in an ELM context
Predictive microbial regulatory behavior (Tagkopoulos et al., 2008)	L2c associative/predictive adaptation benchmark	“Associative adaptive response”	Extinction/overwrite, retention in material setting, single-cell or lineage analysis, and scaffold/interface effects
Optogenetic skeletal muscle-powered biological machines (Raman et al., 2016)	Biohybrid edge case: L1 active actuation; L2–L4 require explicit history evidence	“Optogenetically controlled biohybrid actuation”	Stimulus-history controls, post-training retention, fatigue/damage exclusion, and controller/actuator decoupling
Closed-loop neural cultures in a simulated task environment (Kagan et al., 2022)	Adjacent biohybrid-controller case; attribution belongs to the neural–controller–environment loop, not automatically to a material substrate	“Closed-loop neural biohybrid learning-like performance,” with explicit controller, embodiment, and electrophysiological attribution	Controller-only and open-loop controls, retention/extinction tests, declared reference variable, and material-level substrate attribution
Stimuli-responsive ELM platforms reviewed in Rivera-Tarazona et al. (2021)	L0–L1 by default unless history dependence or persistence is measured	“Stimuli-responsive”; “adaptive” requires stimulus-history evidence	Repeated-stimulus history, persistence, specificity, and component-resolved controls

This exercise is an interpretability stress test, not validation of classification accuracy or inter-reviewer reliability. It should not be read as an assessment of the quality of the cited studies, and absence of a control in this illustrative mapping should not be interpreted as evidence that the original authors failed to perform it unless the focal source was explicitly audited for that item. Supplementary Table S1 therefore records the mapping fields separately—source wording, measured state/output, stimulus or training logic, controls visible in the focal report, unresolved alternatives, proposed ELM-CLAIM level, and calibrated language—and uses “not assessed” where the present synthesis did not verify a field. This avoids converting an illustrative exercise into a false completeness claim.

Four lessons follow. First, abiotic smart materials provide an important boundary condition: shape memory and hysteresis can be real material phenomena without supporting living memory or learning claims. Second, many impressive ELM systems are already strong L1 systems but should not be labeled memory-like or learning-like without additional temporal controls. Third, designed circuit memory is valid but should be named as designed memory and traced to circuit state rather than attributed vaguely to the whole material. Fourth, closed-loop biohybrid systems require controller audits because learning-like behavior may reside in software, living tissue, or coupled interaction. The strongest future ELM-CLAIM applications will involve experiments designed prospectively around the matrix, not retrospective reinterpretation after publication.

## 9 Discussion

### 9.1 What ELM-CLAIM adds

ELM-CLAIM contributes a practical evidential grammar for a rapidly expanding field. Existing reviews classify ELM types, applications, stimuli, and material platforms (Nguyen et al., 2018; Srubar, 2021; Rodrigo-Navarro et al., 2021; Rivera-Tarazona et al., 2021). ELM-CLAIM instead classifies the *claims* made about those systems and maps them to controls and attribution criteria. This shift is important because a single system can support different levels of claim for different outputs.

The framework also makes ELM research more explicitly systems-biological. Rather than treating responsiveness as a single output, it asks how states are encoded, how perturbations reveal causal architecture, how multiscale variables interact, and how models and data can be reused. In this sense, ELM-CLAIM is not only a language-policing tool; it is a design aid for experiments that can actually test adaptive behavior. Crucially, the L0–L5 taxonomy is not a maturity scale or a value hierarchy for materials. It is a calibration of claim strength for a specific measured behavior. A rigorously supported L1 claim may be more scientifically valuable than an under-controlled L4 claim.

### 9.2 Claim calibration is not scientific validation

ELM-CLAIM evaluates whether the strength and attribution of a term are justified by the evidence that is reported. This is narrower than scientific validation. A perfectly calibrated L1 statement can still rest on an underpowered assay, an unrepresentative sample, a poorly controlled fabrication batch, or a model with weak predictive performance. Conversely, a technically strong experiment may be described conservatively at a lower claim level. The checklist should therefore be used alongside, not instead of, ordinary evaluation of experimental design, measurement validity, statistics, replication, model credibility, and causal inference. Correct vocabulary is a guardrail against one class of interpretive error; it is not a certificate of overall robustness.

### 9.3 Avoiding both overclaiming and underclaiming

A common concern is that stringent evidential criteria could suppress exploratory language. ELM-CLAIM is intended to do the opposite. It creates a path by which stronger claims can be earned. Authors who perform persistence assays, stimulus-history controls, recellularization experiments, controller ablations, and reference-state perturbations should be able to make stronger statements than authors who only show an immediate response. Conversely, when controls are missing, the framework gives precise language for provisional or lower-level claims. This improves rather than weakens the paper, because calibrated language is more credible.

### 9.4 Proportional use, study maturity, and experimental burden

The framework is intended to make experimental burden proportional to claim strength, not to impose the full matrix on every ELM study. An early exploratory study may legitimately report an L0 or L1 phenomenon with clear state variables and a small set of matched controls while identifying L2–L4 questions as future work. A mechanistic study should invest more heavily in component perturbation, necessity/sufficiency tests, and temporal controls. A mature validation study that makes learning-like or goal-directed claims should accept a substantially higher burden because those words exclude more alternative explanations.

This proportionality creates an explicit choice for authors: add experiments to support stronger language, or retain a lower-level claim that accurately matches the available evidence. Expensive techniques are not valuable merely because they are expensive. A low-cost orthogonal control that resolves the relevant causal alternative can be more informative than a technically elaborate assay that does not. Conversely, a substituted control should not be accepted simply to reduce burden if it fails to address the evidential function in Section 5.1. The expected burden is therefore driven by the claim and unresolved alternatives, not by a fixed checklist length.

### 9.5 The status of L4 and L5 claims

Learning-like and goal-directed claims require special caution. L4 claims are not excluded from ELM research, but they require acquisition, retention, and exclusion of major confounds. Transfer and extinction provide full L4 support and should be attempted whenever feasible. L4-provisional is only a temporary calibration label for systems in which acquisition and retention are shown but transfer or extinction remain untested; it must never be cited as demonstrated learning-like behavior. L5 claims require still more: an explicitly measured reference variable, a comparator or deviation-detection process, an actuator process, perturbation and manipulation of the reference state, and evidence that reference-state control is generated or maintained by the living/coupled system, not merely by a controller set point. A material that returns to a programmed parameter after disturbance may be an excellent controlled system, but it is not autonomously goal-directed unless the reference condition belongs to the living/coupled architecture in the sense defined above.

### 9.6 Implementation by authors, reviewers, and editors

For authors, ELM-CLAIM can be used prospectively at experimental design stage: choose the intended claim level, then build the corresponding evidential functions into the study. For reviewers, Table 6 provides a direct sequence from vocabulary to state variable, attribution, controls, and revision action. For editors, it provides a way to request targeted revision without rejecting the underlying science: lower the claim, add the specific control that resolves a major alternative, or mark the claim as provisional. The machine-readable checklist (Supplementary Table S2) is intended to make this process reusable without turning it into a pass/fail score. Until inter-reviewer reliability is measured, apparent agreement produced by the form should not be mistaken for validated consistency across laboratories or journals.

**TABLE 6:** How to use ELM-CLAIM in peer review: a six-step workflow.

Step	Reviewer action
1	Identify the strongest adaptive or cognitive-like term used for the specific behavior
2	Locate the measured state variable, stimulus/test protocol, and relevant timescale
3	Assign the lowest component-level attribution consistent with necessity/sufficiency evidence
4	Apply the L0–L5 boundary rules and identify which essential evidential functions are met.
5	For missing ideal controls, evaluate justified substitutes and record residual uncertainty rather than demanding a specific technique
6	Recommend one of three actions: retain the claim, use provisional/lower-level wording, or add a targeted control that resolves a major alternative explanation

### 9.7 Limitations and failure modes

ELM-CLAIM is an author-proposed framework. It has not yet undergone community consensus building, Delphi-style expert elicitation, prospective laboratory validation, or formal inter-reviewer reliability testing. The present published case exercise demonstrates that the categories can be applied, but it cannot establish that different reviewers will apply them reproducibly. Community testing and revision are therefore requirements for maturation of the framework, not optional future refinements.

The framework can fail in several directions. It may be too strict when a biologically meaningful state cannot be cleanly washed out, when transfer or extinction is scientifically undefined, or when a decisive perturbation would destroy the coupled architecture being studied. It may be too flexible if “system-specific” substitutions become a route for accepting weak proxies that do not address the intended causal alternative. It may be difficult to apply reproducibly when timescales are unknown, multiple state variables imply different levels, or reasonable reviewers disagree about which confounds are material. The explicit boundary and substitution rules reduce these problems but do not eliminate judgment.

A further risk is false hierarchy. L0–L5 can be misread as a ladder of system quality, encouraging authors to pursue high labels rather than strong science. The framework instead ranks evidential commitments for a particular behavior. Another risk is over-formalization: completion of a checklist may create an appearance of rigor while measurement validity, statistics, fabrication variability, or model credibility remain weak. The distinction between claim calibration and validation above is intended to prevent such box-ticking.

Finally, controls can impose substantial cost, time, and material burden, especially in primary-cell, multicellular, ecological, or long-duration ELMs. The proportional substitution rule is intended to prevent technically privileged platforms from becoming the implicit norm. Nevertheless, when no feasible control addresses an essential alternative, ELM-CLAIM deliberately favors a lower or provisional claim. The framework also does not settle philosophical debates about minimal cognition, consciousness, or sentience; it separates empirical thresholds from those broader interpretations.

### 9.8 Future validation

The next step is empirical validation of the framework itself. Three tests are especially important. First, multiple independent reviewers should classify the same set of ELM abstracts and full papers to estimate inter-reviewer reliability. Second, laboratories should design prospective L2–L4 experiments using the matrix and report which controls are feasible. Third, journals or special issues could pilot the checklist as optional supplementary information for papers using adaptive or cognitive-like vocabulary. A fourth test should quantify the additional experimental and reporting burden created at each claim level, including whether proportional substitutions reduce burden without reducing reviewer agreement. Only after such evidence and broader community deliberation should ELM-CLAIM be described as a community-refined standard rather than an author-proposed framework.

## 10 Conclusion

Engineered living materials make possible a new class of adaptive hybrid systems in which cells, scaffolds, interfaces, morphology, environments, and controllers jointly determine material function. This novelty justifies ambitious language, but only when the language is operationally grounded. ELM-CLAIM proposes one route to that grounding. It defines ELM behavior, supplies a six-level claim taxonomy, maps claims to tiered evidential functions, formalizes attribution across system components, aligns reporting with reproducibility and model-credibility practices, and illustrates its use on published cases.

The central message is simple: do not forbid adaptive vocabulary, but make it earn its evidential status. A system may be responsive, actively regulated, adaptive, memory-like, learning-like, or goal-directed; each term becomes more powerful when its measurement, timescale, mechanism, and attribution are clear. If future community testing shows that the levels and decision rules can be applied reliably, ELM-CLAIM may help transform adaptive-language claims from evocative metaphors into more explicit, testable, and reproducible systems-level scientific assertions.

## Data Availability

The original contributions presented in the study are included in the article/Supplementary Material, further inquiries can be directed to the corresponding author.
